# Molecular Mechanisms Regulating LPS-Induced Inflammation in the Brain

**DOI:** 10.3389/fnmol.2016.00019

**Published:** 2016-03-08

**Authors:** Olena Lykhmus, Nibha Mishra, Lyudmyla Koval, Olena Kalashnyk, Galyna Gergalova, Kateryna Uspenska, Serghiy Komisarenko, Hermona Soreq, Maryna Skok

**Affiliations:** ^1^Laboratory of Cell Receptors Immunology, O. V. Palladin Institute of BiochemistryKyiv, Ukraine; ^2^The Edmond and Lily Safra Center of Brain Science and The Alexander Silberman Institute of Life Sciences, The Hebrew University of JerusalemJerusalem, Israel

**Keywords:** inflammation, brain, **α**7 nicotinic acetylcholine receptor, acetylcholine esterase, microRNA, antibody

## Abstract

Neuro-inflammation, one of the pathogenic causes of neurodegenerative diseases, is regulated through the cholinergic anti-inflammatory pathway via the α7 nicotinic acetylcholine receptor (α7 nAChR). We previously showed that either bacterial lipopolysaccharide (LPS) or immunization with the α7(1–208) nAChR fragment decrease α7 nAChRs density in the mouse brain, exacerbating chronic inflammation, beta-amyloid accumulation and episodic memory decline, which mimic the early stages of Alzheimer’s disease (AD). To study the molecular mechanisms underlying the LPS and antibody effects in the brain, we employed an *in vivo* model of acute LPS-induced inflammation and an *in vitro* model of cultured glioblastoma U373 cells. Here, we report that LPS challenge decreased the levels of α7 nAChR RNA and protein and of acetylcholinesterase (AChE) RNA and activity in distinct mouse brain regions, sensitized brain mitochondria to the apoptogenic effect of Ca^2+^ and modified brain microRNA profiles, including the cholinergic-regulatory CholinomiRs-132/212, in favor of anti-inflammatory and pro-apoptotic ones. Adding α7(1–208)-specific antibodies to the LPS challenge prevented elevation of both the anti-inflammatory and pro-apoptotic miRNAs while supporting the resistance of brain mitochondria to Ca^2+^ and maintaining α7 nAChR/AChE decreases. In U373 cells, α7-specific antibodies and LPS both stimulated interleukin-6 production through the p38/Src-dependent pathway. Our findings demonstrate that acute LPS-induced inflammation induces the cholinergic anti-inflammatory pathway in the brain, that α7 nAChR down-regulation limits this pathway, and that α7-specific antibodies aggravate neuroinflammation by inducing the pro-inflammatory interleukin-6 and dampening anti-inflammatory miRNAs; however, these antibodies may protect brain mitochondria and decrease the levels of pro-apoptotic miRNAs, preventing LPS-induced neurodegeneration.

## Introduction

Neuro-inflammation accompanies and often precedes the development of neurodegenerative pathologies such as Parkinson’s and Alzheimer’s diseases (AD; Wee Yong, 2010) and might be one of the pathogenic factors for neurodegeneration (Chung et al., 2010; Heppner et al., 2015). However, the molecular mechanisms linking inflammatory reaction to degeneration in the brain are poorly understood. One of the cross-points may include nicotinic acetylcholine receptors of the α7 subtype (α7 nAChRs), which are involved in both cholinergic anti-inflammatory and pro-survival intracellular pathways (Ji et al., 2014; Báez-Pagán et al., 2015; Terrando et al., 2015; Truong et al., 2015).

A number of studies point out the involvement of α7 nAChRs in pro-survival cell signaling, engaging the PI3K/Akt signaling pathway (Parada et al., 2010; Yu et al., 2011; Cucina et al., 2012; Huang et al., 2012; Cui et al., 2013). Such signaling was shown to protect cultured brain cells from apoptosis induced by various apoptogenic agents (Parada et al., 2010). Moreover, α7 nAChRs were found to regulate mitochondrial permeability transition pore formation and release of apoptogenic factors like cytochrome (cyt *c*) and therefore, to control the mitochondrial-mediated pathway of apoptosis (Gergalova et al., 2012, 2014).

In addition, α7 nAChR directly interacts with the amyloid-beta (Aβ) precursor protein (Ikonomovic et al., 2009), which favors its normal processing by α-secretase (Kim et al., 1997; Qi et al., 2007) and promotes internalization of processed Aβ forms (Wang et al., 2000; Parri and Dineley, 2010). Consequently, the decrease of α7 nAChR density on the plasma membrane impairs Aβ metabolism and favors accumulation of extracellular Aβ (1–42) (Gouras et al., 2005; Dziewczapolski et al., 2009).

Previously, we found that regular injections of bacterial lipopolysaccharide (LPS) decreased the density of α7 nAChRs in the brain and brain mitochondria of mice and reduced nucleated cell numbers in the hippocampus and striatum, while stimulating astrocytosis, accumulation of Aβ (1–42) peptides and episodic memory decline—symptoms characteristic of the early stages of AD (Lykhmus et al., 2015). Antibodies, generated *in vivo* by immunization of mice with recombinant extracellular domain of α7 nAChR subunit, α7(1–208), facilitated symptoms similar to those induced by LPS but did not cause degeneration in the brain of mice (Lykhmus et al., 2015), indicating the involvement of specific regulatory processes.

Another important regulator of cholinergic signaling is acetylcholinesterase (AChE), the levels of which decrease during inflammation, increasing acetylcholine levels and stimulating the anti-inflammatory pathway (Soreq, 2015). Acetylcholine was shown to attenuate the release of pro-inflammatory cytokines, like IL-1 or TNFα, by peritoneal monocytes and macrophages in response to bacterial endotoxin—LPS through α7 nAChRs (Borovikova et al., 2000). This phenomenon, first described in 2000 and called “Cholinergic Anti-Inflammatory Pathway”, was further observed in many organs and tissues including the brain (de Jonge and Ulloa, 2007; Tyagi et al., 2010; Thomsen and Mikkelsen, 2012; Ji et al., 2014; Báez-Pagán et al., 2015; Egea et al., 2015; Truong et al., 2015). AChE expression has been shown to be regulated by microRNAs (miRNAs), small non coding RNAs suppressors of entire pathways of gene expression (Chen et al., 2004; Soreq and Wolf, 2011). MiRNA-132 is reported to increase during inflammation in many tissues (Maharshak et al., 2013; Shaltiel et al., 2013; Nadorp and Soreq, 2015) and is validated to target AChE further to potentiate cholinergic anti-inflammatory pathway (Shaked et al., 2009; Soreq and Wolf, 2011).

The present study was aimed to reveal the molecular mechanisms underlying the LPS and antibody effects in the brain, using a model of acute LPS-induced inflammation with or without α7-specific antibody injections. Specifically, we studied the involvement of α7 nAChRs in brain inflammation and mitochondrial apoptosis, measured changes in AChE levels with inflammation and profiled brain miRNAs under exposure to LPS, LPS and α7-specific antibody (Ab α7) or nicotine. Our findings indicate that LPS down-regulates α7 nAChR and AChE in the brain; exacerbates the mitochondrial pathway of apoptosis and changes brain miRNAs in favor of pro-apoptotic and anti-inflammatory ones. Inversely, the antibody supports the integrity of brain mitochondria and attenuates the LPS-induced pro-apoptotic miRNAs up-regulation while stimulating pro-inflammatory signaling and preventing the LPS-induced elevation of the anti-inflammatory miRNA-132/212 (Shaked et al., 2009; Shaltiel et al., 2013; Soreq, 2015).

## Materials and Methods

### Animals and Reagents

Female 3 months old C57BL/6J mice were housed in a quiet, temperature-controlled room (22–23°C) in the animal facility of the O.V. Palladin Institute of Biochemistry and were provided with water and dry food pellets *ad libitum*. Mice were sacrificed by cervical dislocation to remove the brain. All procedures of this study conformed to the guidelines of the Animal Care and Use Committee of Palladin Institute and were approved by the IACUC Protocol 1/7–421.

All reagents were of chemical grade and were purchased from Sigma-Aldrich unless specially indicated. Antibodies against α7(1–208), α7(179–190), α3(181–192), α4(181–192), β2(190–200) and β4(190–200) nAChR fragments were obtained and characterized by us previously (Skok et al., 1999; Koval et al., 2004; Lykhmus et al., 2011).

Glioblastoma U373 cells (ATCC HTB17–1055) were a kind gift of Prof. A.V.Ryndich from the Institute of Molecular Biology and Genetics in Kyiv.

### Animal Treatment and Samples Preparation

Mice were intra-peritoneally injected with LPS (30 μg/mouse in PBS) on days 0 and 2 (groups 1 and 2). Group 2 mice were additionally intravenously injected with α7(1–208)-specific antibodies (200 μg/per mouse in saline) on days 0, 1 and 2. Group 3 mice received nicotine in the drinking water (200 μl/l) for either 3 days or 1 month and the 4th group was intact (Ctrl).

On the 3rd day of each treatment, mice were sacrificed; their brains were removed, homogenized and fractionated into primary pellets (nuclei, cell debris) and mitochondria (additionally pelleted from the supernatant) by a standard procedure of differential centrifugation (Gergalova et al., 2012). To quantify nAChR subunits, both mitochondria and primary pellets were lysed in detergent-containing buffer. Live mitochondria were further examined for functional activities.

In another set of experiments, the brains of similarly treated mice were divided into two halves (hemispheres) and each half was dissected into Hippocampus, Striatum/Thalamus, Cerebellum and Frontal cortex. The one half sections were homogenized under liquid nitrogen and used for RNA extraction. Sections of the 2nd half were homogenized; lysed in detergent-containing buffer (0.01 M Tris-HCl, pH 7.4, 1 M NaCl, 1 mM EGTA, 1% Triton X-100) for 45 min on ice and centrifuged at 13,000 rpm. The resulting supernatant was used for quantifying AChE activity and α7 nAChR protein. Protein content was measured with the BCA kit (Thermo Scientific, France).

### Cytochrome *c* (cyt *c*) Release Assay with Live Mitochondria

Cyt *c* release from isolated mitochondria was measured as described previously (Gergalova et al., 2012). Briefly, purified mitochondria (120 μg of protein per ml) were incubated with different doses of CaCl_2_ with or without the α7 nAChR agonist PNU282987 (30 nM) for 2 min at room temperature and were immediately pelleted by centrifugation. The supernatants were tested for the presence of cyt *c* by a sandwich ELISA assay.

### Quantifying nAChR Subunits in the Brain or Mitochondria Preparations

The assay was performed as described Lykhmus et al. (2015). Briefly, immunoplates (NUNC, MaxiSorp) were coated with rabbit α7(1–208)-specific antibody (20 μg/ml), blocked with 1% BSA, and the tested preparations were applied into the wells (1 μg of protein per 0.05 ml per well) for 2 h at 37°C. The plates were washed with water and the second biotinylated α3(181–192), α4(181–192), α7(179–190), β2(190–200) or β4(190–200)-specific antibody was applied for additional 2 h being revealed with Streptavidin-peroxidase conjugate and *o*-phenylendiamine-containing substrate solution.

### RT-PCR for **α**7, AChE and microRNA Transcripts

RNA extraction from tissue samples was carried out using Trizol (Sigma, NY, USA) as described Shaked et al. (2009). RNA concentration and integrity were determined spectrophotometrically and by electrophoresis, respectively. RNA samples (500 ng) were reverse transcribed using the Quanta cDNA synthesis kit for mRNA and miRNA as per the manufacturer’s (Quanta Biosciences) protocol. Real-time RT-PCR was performed using the ABI prism 7900 HT and SYBR green master mix (Quanta Biosciences). For miRNA transcripts, PerfeCTa microRNA assay primers (Quanta Biosciences) were used. Results were normalized to the expression of snoRD47 and actin for miRNA and mRNA respectively. Further all the results were normalized to respective regional control. The following primers were used for: AChE-S Forward (F): CTGAACCTGAAGCCCTTAGAG, Reverse (R): CCGCCTCGTCCAGAGTAT; nAChR7, F: CACATTCCACACAACGTCTT, R: AAAAGGGAACCAGCGTACATC; actin F: CCACACCCGCCACCAGTT, R: TACAGCCCGGGGAGCAT. Fold change values for both miRNAs and mRNAs were calculated using the ΔΔCt method.

### RNA-seq Library Preparation and Sequencing

Libraries for next generation sequencing (NGS) were prepared from whole brain RNA using TruSeq Small RNA Library Prep Kit as per the manufacturer’s protocol. A total of four libraries (pooled from four animals of each group) were prepared from RNA of four groups. Briefly, the total 600 ng of RNA samples were hybridized with Trueseq Adaptor Mix which is a set of oligonucleotides with a single-stranded degenerate sequence at one end and a defined sequence required for Miseq sequencing at the other end. The Adaptor Mix constrains the orientation of the RNA in the ligation reaction such that hybridization with it yields template for sequencing from the 5^′^ end of the sense strand. After hybridization, the adaptors are ligated to the small RNA molecules using the Ligation Enzyme Mix, which is a mixture of an RNA Ligase and other components. Ligation requires an RNA molecule with a 5^′^-monophosphate and a 3-hydroxyl end; therefore, most small RNAs can participate in this reaction, and intact mRNA molecules with a 5^′^-cap structure are excluded. Next, the small RNA population with ligated adaptors of each sample was reverse transcribed, to generate cDNA libraries. Treatment with RNaseH followed, to digest the RNA from RNA/cDNA duplexes and to reduce the concentration of un-ligated adaptors and adaptor by-products. The cDNA libraries were amplified using bar coded primer sets and 15–18 cycles of PCR. The amplified cDNA libraries were cleaned up and size selected from gel—PCR products of 105–150 bp were isolated, corresponding to inserts derived from the small RNA population. The amplified cDNA libraries generated were there after used for Miseq sequencing.

miRNA pathway analysis was performed using the microT-CDS tool available through Diana Tools ^1^.

### Determination of AChE Activity

The acetylcholine hydrolyzing activity of AChE was measured by the Ellman’s assay (Ellman et al., 1961) as described Arbel et al. (2014). Briefly, samples were diluted 1:5 with 0.2 M phosphate buffer pH 7.4. Ellman’s reagent was added to each sample, the mixture was incubated at RT for 20 min under darkness, acetylthiocholine iodide was added and absorbance of the corresponding plate wells at 405 nm was monitored with Stat-Fax 2000 ELISA Reader (Advanced Technologies, IL, USA) at 15 time points with 2 min intervals. AChE activity was calculated based on the concentration of the resultant 5-thio-2-nitrobenzoate anion (ε_405_ = 13.6 M^−1^ cm^−1^); taking into account the average OD increment per minute and protein concentration in the sample.

### U373 Culturing, Staining and Imaging

Glioblastoma U373 cells were cultured in RPMI 1640 medium supplemented with 20 mM L-glutamine, 20 mM HEPES, penicillin-streptomycin mixture and 10% fetal calf serum. For microscopy studies, the cells were attached onto glass slides, 3 × 10^4^ per slide, in complete medium for 3 h and were incubated with biotinylated α7(179–190)-specific antibody (0.06 mg/ml) and MitoTracker Green (Invitrogen, USA) for 100 min at 37°C. Cells were fixed with 4% paraformaldehyde and treated with Streptavidin-Cychrome3 conjugate to visualize the biotinylated antibody, followed by washes with PBS and water, embedding in MOWIOL-DABCO and examination in a Zeiss LSM 510 Meta confocal laser scanning microscope.

### Measuring IL-6 Produced by U373 Cells

U373 cells (3 × 10^5^ per ml) seeded into the wells of 96-well plates were cultured with LPS (clone 055 B5; 1 μg/ml) or α7(179–190)-specific antibody (10 μg/ml) in the presence or absence of the following kinase inhibitors: SB202190 (10 μM, p38 inhibitor), KN62 (1 μM, CaKMII inhibitor), PP1 (10 μM, Src kinase inhibitor), bisindolylmaleimide (50 nM, protein kinase C inhibitor) and Wortmaninn (1 μM, phosphatidylinositol-3-kinase inhibitor) for 24 h. The IL-6 concentration in the supernatants was detected using Diaclone test system as per the manufacturer’s instructions.

### Statistical Analysis

Each experiment has been performed in minimum 7 mice and ELISA assays for each mouse have been performed in triplicates. The mean values for individual mice were used for statistical analysis using Student’s *t*-test. The data are presented as M ± SE; *p < 0.05; **p < 0.005.

## Results

### Both LPS and **α**7(1–208)-Specific Antibodies Modulate nAChR Composition in the Brain

**Figure 1:**
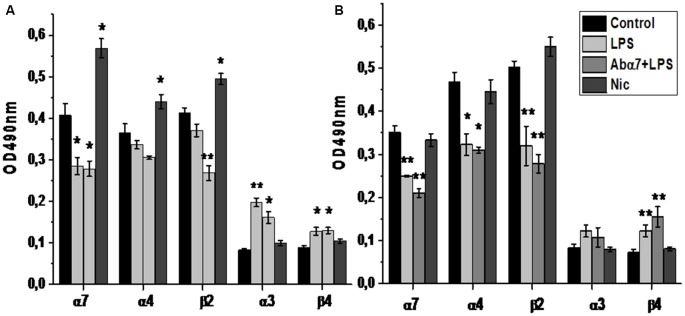
**The level of α3, α4, α7, β2 and β4 nAChR subunits in the whole brain preparations (A) or brain mitochondria (B) of mice treated with lipopolysaccharide (LPS), α7(1–208)-specific antibody + LPS (Abα7 + LPS) or nicotine (1 month, Nic) compared to non-treated mice (Control).** *p < 0.05; **p < 0.005 compared to Control (*n* = 4).

**Figure 2:**
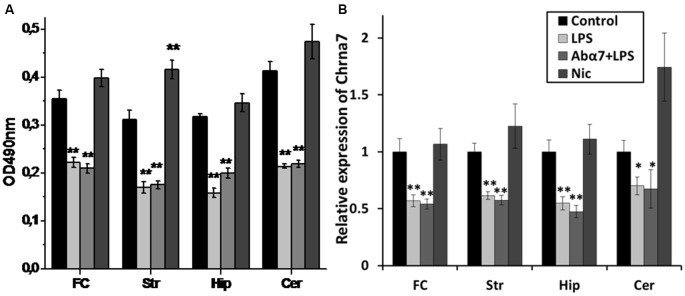
**Modified levels of α7 nAChR protein (A) or RNA (B) in various brain regions of mice treated with LPS, α7(1–208)-specific antibody + LPS (Abα7 + LPS) or nicotine (Nic) compared to non-treated mice (Control).** FC—frontal cortex, Str—striatum, Hip—hippocampus, Cer—cerebellum *p < 0.05; **p < 0.005 compared to Control (*n* = 8).

Sandwich ELISA performed with whole brain preparations demonstrated that LPS treatment decreased the level of α7 nAChR subunits while increasing the α3 and β4 subunits. Injections of α7(1–208)-specific antibodies additionally decreased the α4 and β2 subunits (Figure 1A). Nicotine (3 days) up-regulated both α7 and α4β2 nAChRs and did not influence α3β4 ones. LPS and LPS plus α7(1–208)-specific antibody, but not nicotine, decreased α7 and α4β2 nAChRs and non-significantly increased α3β4 ones in mitochondria preparations (Figure 1B). LPS treatment decreased both α7 RNA and protein in the frontal cortex, striatum, hippocampus and cerebellum. The α7(1–208)-specific antibody did not modify the effect of LPS on α7 nAChR RNA or protein expression. Nicotine slightly increased the α7 protein in the striatum but did not affect its RNA level (Figures 2A,B).

### LPS and **α**7(1–208)-Specific Antibodies Modulate Brain AChE Expression and Activity

**Figure 3:**
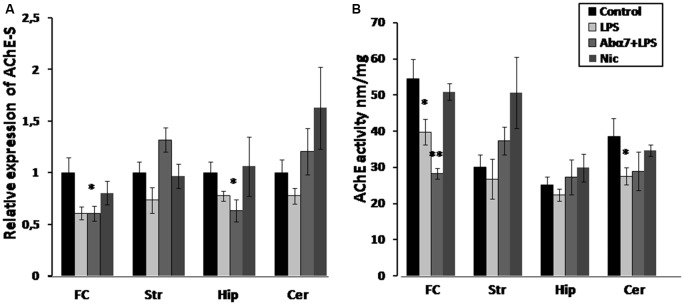
**Transcript levels (A) and enzyme activity (B) of acetylcholinesterase-S (AChE-S) in various brain regions of mice treated with LPS, α7(1–208)-specific antibody + LPS or nicotine (3 days) compared to non-treated mice.** Designations are similar to those in Figure 2. *p < 0.05; **p < 0.005 compared to Control (*n* = 8).

LPS exposure significantly decreased the levels of the “synaptic” AChE-S variant (Soreq and Seidman, 2001) in the frontal cortex and non-significantly in the striatum, hippocampus and cerebellum that was interpreted as a tendency to decrease. The antibody additionally decreased AChE-S RNA in the hippocampus. Nicotine (3 days) caused non-significant up-regulation of AChE-S in the striatum (Figure 3A). Enzyme activity measurements demonstrated decreased AChE activity in the frontal cortex and cerebellum and a tendency to decrease in the striatum and hippocampus under the effect of LPS. The antibody accentuated the LPS effect in the frontal cortex, while nicotine tended to increase AChE activity in striatum (Figure 3B). A correlation between AChE-S expression and activity was observed in the frontal cortex, cerebellum and striatum of individual mice (Pearson coefficient being from 0.80–0.99; data not shown).

### LPS and **α**7(1–208)-Specific Antibodies Modify the Reaction of Brain Mitochondria to Apoptotic Stimuli

**Figure 4:**
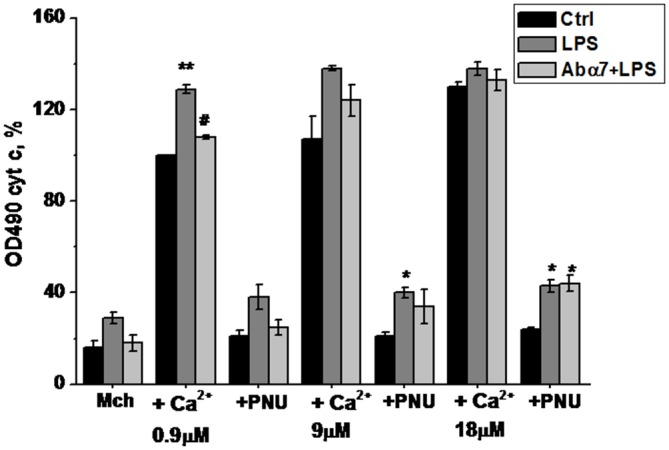
**Cytochrome *C* release from the brain mitochondria of mice treated with LPS or α7(1–208)-specific antibody + LPS compared to untreated mice under the effect of Ca^2+^ and/or PNU282987.** Designations are similar to those in Figure 1. Shown are the normalized data of three independent experiments, 3–4 mice in each. *p < 0.05; **p < 0.005 compared to Control; ^#^*p* < 0.05 compared to LPS.

Live mitochondria isolated from the brains of LPS-treated mice released more cyt *c* in response to 0.9 and 9.0 μM Ca^2+^ and became less sensitive to the normalizing effect of the α7-specific agonist PNU282987 than mitochondria of non-treated animals (Figure 4). Moreover, mitochondria of LPS-treated mice released some cyt *c* without any Ca^2+^, reflecting their unstable (pre-apoptotic) state. The α7(1–208)-specific antibodies decreased the LPS-induced cyt *c* release from mitochondria at low Ca^2+^ doses and facilitated the normalizing effect of PNU282987.

### The Effect of LPS and **α**7(1–208)-Specific Antibody on the microRNA Spectrum in the Brain

To delineate the differentially expressed miRs in brain during inflammation we performed whole brain miR-deep sequencing analysis. A total of 24, 249, 523 sequencing reads were obtained. All the reads (50 bp long) were subjected to trimming of the tag end terminal base pairs and P1 start adapter (Miseq miRNA reverse primer sequences) using CLC genomics workbench V7.0 (CLC Inc, Aarhus, Denmark). The remaining reads (1, 88, 986) obtained were aligned against the mouse miRNA genome (miRBase release 20) and Ensemble mouse database (GRCm38) for non coding RNA. Through the annotation and merge counts, only reads longer than 15 bases were analyzed. Match parameters included for mature length variants (IsomiRs)-additional two upstream and downstream bases, and two missing upstream and downstream bases, and maximum allowed mismatches two using standard specific alignment protocol. On average, 70% (range 76.6–50.6%) of the sequences of all annotated reads were mapped and 85% (range 90.4–76.7%) of miRBase genes were detected. Of the mapped reads, on average 11% (range 12.2–10.8%) had perfect match to the aligned genes, 54% (range 54.5–55.2%)-one mismatch, 23% and (range 21.7–24.7%) two mismatches. Mapping to Ensemble mouse database (GRCm38) yielded mapping to 10.5% (10–11%) of all annotated database sequences on average across libraries. Preference was given to miRBase, so the database which was not mapped to miRBase is mapped to this. Of them, 55.6% (range 55.6–37.3%) exhibited perfect match to the reference sequences, 39.6% (range 39.6–51.5%) with one mismatch, and 8.3 (8.3–11.2%) two mismatches. These alignments yielded an average of 2.06% aligned reads of the total number of reads (with a minimum of 0.2% and a maximum of 3.16%). Annotated samples were grouped by both precursor and mature sequence identity. Overall, a top 44 mature miRs that exhibited count of at least 30 per million reads in at least one sample were analyzed for differential expression between the different experimental conditions. For fold change analysis the above identified miRs of different groups were normalized to control group and that were 1.5 up-regulated or 0.5 fold down-regulated were identified as uniquely regulated miRs.

**Figure 5:**
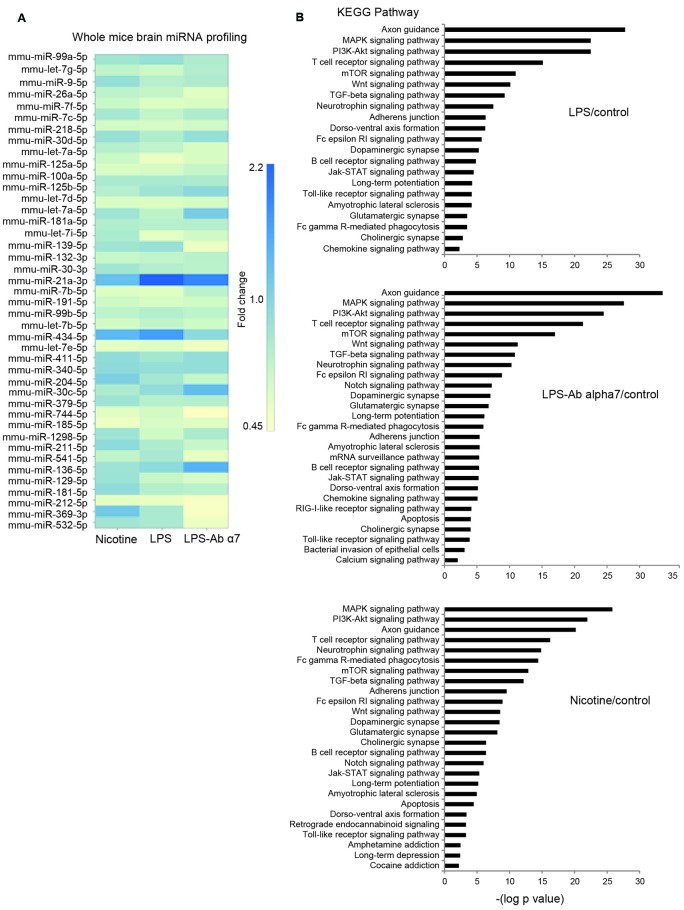
**(A)** Fold changes in the levels of 44 differentially expressed miRs in the whole brain preparations of mice treated with LPS, α7(1–208)-specific antibody + LPS or nicotine (1 month) compared to untreated mice (Control), **(B)** KEGG pathway analysis of predicted signaling pathways involved.

The wide screening of the brain RNA for the expression of 44 miRs demonstrated that the cluster of the highest expression contained miRs-99a, let7g and 9, that the cluster of medium expression included miRs-26a and let-7f, and that other miRs were of quite low or very low expression. The miR-21 and miR-434 were observed twofold up-regulated and let-7a-1 0.5 fold down-regulated. As shown in Figure 5A, all types of treatment (LPS, LPS + α7(1–208)-specific antibody or nicotine) influenced the level of many of them. MiR-99a was obviously up-regulated by both nicotine and LPS and the effect of LPS was withdrawn by the antibody. In contrast, miR-let7g was down-regulated by LPS and, less, nicotine but the effect of LPS was again withdrawn by the antibody. MiR-9 was up-regulated by nicotine and much less affected by the LPS; however, again, the antibody effect was opposite to that of LPS. MiRs-26a and let-7f were down-regulated in all groups of treated mice; the antibody obviously aggravated the LPS effect for miR-26a. Among other miRs, of considerable interest are miR-21a and miR-434: both of them were up-regulated in all treated mice, mostly by LPS, and the antibody prevented this effect more or less efficiently. In whole, the antibody obviously attenuated the LPS effect in 16 miRs tested and aggravated its effect in six miRs. The effects of nicotine and LPS were of similar direction for six miRs (miR-26, let-7f, let-7c, miR-30, miR-21a and miR-434) and showed an opposite impact for other four miRs (miR-9, let-7j, miR-218 and miR-125).

After miRNA differential expression analysis, we performed KEGG pathways analysis of differentially regulated miRNA of each group exposed to either LPS or LPS and Ab α7 or nicotine (Figure 5B). After removing redundant terms, our findings pointed that all pathways involved in LPS exposure were included in LPS and Ab α7 exposure group. In addition, this group was also associated with Notch signaling, apoptosis, mRNA surveillance, bacterial invasion and calcium signaling pathways. Similarly, nicotine group included all pathways involved in LPS inflammation; in addition it was associated with addiction pathways like Cocaine, Amphetamine, Retrograde endocannabinoid signaling, apoptosis and Notch signaling. Common KEGG pathways found in all treatment groups are either neuronal or inflammatory pathways which included axon guidance, MAPK signaling pathway, PI3K-Akt signaling pathway, T cell receptor signaling pathway, Neurotrophin signaling pathway, mTOR signaling pathway, TGF-beta signaling pathway, Wnt signaling pathway, Fc gamma R-mediated phagocytosis, Adherence junction, Glutamatergic synapse, Long-term potentiation, Dopaminergic synapse, Amyotrophic lateral sclerosis (ALS), Fc epsilon RI signaling pathway, Dorso-ventral axis formation, Cholinergic synapse, B cell receptor signaling pathway, Jak-STAT signaling pathway, Toll-like receptor signaling pathway, chemokine signaling pathway.

### qRT PCR Analysis of miRNA-132/212 in Different Regions of the Brain

**Figure 6:**
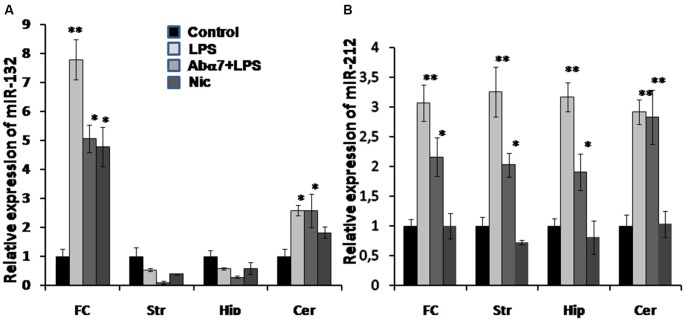
**Transcript levels of miR-132 (A) and miR-212 (B) in various brain regions of mice treated with LPS, α7(1–208)-specific antibody + LPS or nicotine (3 days) compared to untreated mice.** Designations are similar to those in Figures 1– 5. *N* = 5–8/group. *p < 0.05; **p < 0.005 compared to Control.

To further understand the down-regulation in expression of the cholinergic α7 nAChR (global) and AChE-S (brain region specific) genes, we performed qRT PCR analysis for quantifying the expression of AChE-S targeting miRs: miR-132 and its co-clustered miR-212 in the above mentioned four brain regions. We observed significant LPS-induced increases in the expression levels of miR-212 in all of the tested brain regions, whereas miR-132 showed region-specific (frontal cortex and cerebellum) increases in its expression, correlating to AChE-S expression (Figures 3A,B vs. Figure 6). The antibody tended to cause decreases in miR-132 expression that was up-regulated by LPS in the frontal cortex, hippocampus and striatum. In comparison, the antibody treatment clearly prevented LPS-induced miR-212 up-regulation in all brain regions except cerebellum (Figure 6B). Nicotine (3 days) failed to significantly affect either miR-132 or miR-212 expression.

### LPS and **α**7(1–208)-Specific Antibodies Stimulate IL-6 Production by U373 Cells through a Similar Signaling Pathway

**Figure 7:**
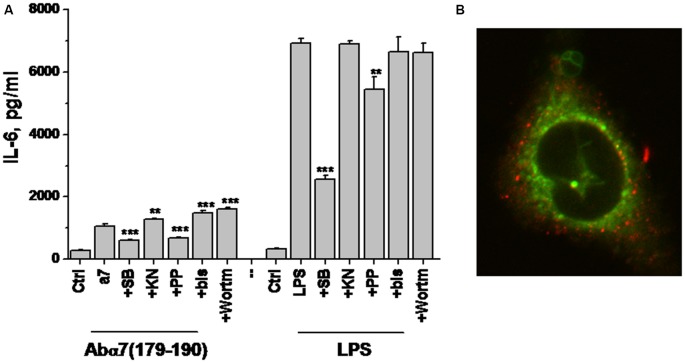
**IL-6 production by U373 cells stimulated with either α7(179–190)-specific antibody or LPS in the presence or absence of various kinase inhibitors (A) and confocal image of U373 cell with internalized α7-specific antibody (red) additionally stained with MitoTracker (green; B).** SB—SB202190 (p38 inhibitor), KN—KN62 (CaKMII inhibitor), PP—PP1 (Src kinase inhibitor), bis—bisindolylmaleimide (protein kinase C inhibitor), Wortm—Wortmaninn (phosphatidylinositol-3-kinase inhibitor). **p < 0.005; ***p < 0.0005 compared to the effect of LPS or α7-specific antibody alone.

Previously we demonstrated that α7(1–208)-specific antibodies and even more, α7(179–190)-specific antibodies stimulated IL-6 production in U373 cells via a p38-dependent pathway (Kalashnyk et al., 2014). To test if similar or different mechanisms are involved in LPS- or antibody-stimulated IL-6 production, we tested the effect of various kinase inhibitors on these consequences. As shown in Figure 7A, the antibody stimulated much weaker IL-6 production compared to LPS in U373 cells; however, both LPS-stimulated and antibody-stimulated IL-6 levels were significantly decreased in the presence of p38 and Src kinase inhibitors, suggesting the involvement of a similar Src/p38-dependent signaling pathway.

To study if internalized α7-specific antibodies can bind mitochondria, we allowed their internalization by U373 cells for 100 min, followed by MitoTracker Green staining. Confocal microscopy showed no overlapping staining for the antibody and MitoTracker (Figure 7B); therefore, the internalized antibody seemed not to bind mitochondria.

### Discussion

The influence of α7 nAChR signaling on pro-inflammatory cytokines production is well documented (de Jonge and Ulloa, 2007; Kalashnyk et al., 2014). Our data support, deepen and extend these findings, showing that inflammation regulates α7 nAChR and AChE-S expression and changes miRNA profiles in the brain. Intraperitoneal LPS injections resulted in down-regulation of brain α7 nAChR and AChE-S RNAs and protein levels. AChE down-regulation was accompanied by up-regulation of its targeting miRNA-132 (Shaked et al., 2009; Shaltiel et al., 2013) in the frontal cortex and cerebellum of LPS-treated mice. This region-specific inter-related regulation of miRNA-132/AChE is compatible with the involvement of the cluster harboring miRNA-132/212 in the resolution of inflammation (Nahid et al., 2011; Rao et al., 2015). The general up-regulation of miRNA-212 in all studied brain areas and its significant inhibition by the α7-specific antibody in the frontal cortex, striatum and hippocampus suggests its specific involvement in inflammation-related mechanisms, different from those regulated by miRNA-132. In general, this data indicates that the LPS-induced inflammatory reaction also stimulated the anti-inflammatory cholinergic pathway by leading to consequent increases in ACh levels due to down-regulated AChE expression and activity. However, inhibiting the expression of α7 nAChRs (which presumably mediate the anti-inflammatory effect of ACh) makes inefficient this LPS-stimulated anti-inflammatory response.

Down-regulation of α7 nAChRs was accompanied by the increase of α3β4 nAChRs, similarly to what we observed in α7^−/−^ mice or in mice chronically treated with LPS (Lykhmus et al., 2011, 2015). This means that α7 to α3β4 nAChRs substitution is an established mechanism, which could possibly be due to the chromosomal arrangement of nAChR subunit genes; and that a rather short LPS influence (3 days) is sufficient to stimulate this gene expression exchange. The antibody additionally decreased α4 and β2 protein in the brain, possibly due to the cross-reactivity of α7(1–208)-specific antibodies with the homologous α4 subunit, resulting in α4β2 receptors internalization and degradation. Nicotine treatment did not cause significant changes in the nAChR or AChE expression but up-regulated α4, α7 and β2 proteins in the whole brain that is in accordance with the suggested chaperon-like activity of nicotine (Sallette et al., 2005). We conclude that LPS and α7(1–208)-specific antibodies manipulate the molecular components of the brain’s cholinergic anti-inflammatory pathway.

Similarly to the chronic LPS treatment (Lykhmus et al., 2015), short-term LPS exposure decreased the level of mitochondrial α7 nAChRs and made the brain mitochondria more sensitive to Ca^2+^. The α7-specific antibody prevented the additional cyt *c* release from mitochondria and, therefore, supported their resistance to apoptogenic influence. Since α7(1–208)-specific antibodies attenuated Ca^2+^-stimulated cyt *c* release from isolated mitochondria (Gergalova et al., 2014), we hypothesized that the antibody could penetrate the brain cells to directly affect mitochondria, as it was recently suggested for anti-mitochondrial antibodies in patients with *Pemfigus vulgaris* (Chernyavsky et al., 2015). However, in our experiments, no binding of internalized antibody with mitochondria was observed in U373 cells (Figure 7B) assuming the involvement of indirect molecular mechanism, possibly, including miRNAs.

Over a decade many miRNAs showed functional involvement in neuro-inflammatory mechanisms (Soreq and Wolf, 2011; Maharshak et al., 2013; Nadorp and Soreq, 2015). Analysis of the present data suggests the involvement of miRNAs in regulating the brain cell survival under the effect of LPS and α7-specific antibodies. The brain-abundant miRNA-9, which was down-regulated by LPS exposure, inhibits the expression of a proapoptotic Bcl-2L11 found in the outer mitochondrial membrane (Li et al., 2014); its potential pro-apoptotic effect was largely avoided by the antibody. Another brain-abundant miRNA-99 regulates pro-survival Akt/mTOR signaling (Jin et al., 2013); its up-regulation with LPS was expected to decrease the brain cell viability; whereas the α7(1–208)-specific antibody limited this effect. Likewise, let-7g suppresses the expression of the anti-apoptotic protein Bcl-xL (Wu et al., 2015); therefore, its down-regulation by LPS might be pro-apoptotic, whereas the antibody treatment inhibited this effect. In whole, LPS exerted anti-survival changes in multiple brain miRNAs, and the α7-specific antibody could antagonize part of them, providing a tentative explanation for the protective antibody effect in mitochondria found here. According to our previously published data, α7 nAChR is located in the outer mitochondria membrane and its activation stimulates intramitochondrial Pi3K/Akt signaling pathway (Gergalova et al., 2012, 2014), that is in good agreement with the present results. The pro-survival antibody effect also explains the data of Kamynina et al. (2013) and Lykhmus et al. (2015), where α7-specific antibodies exerted neuroprotection in certain models of AD.

In addition to their involvement in inflammation-related processes, both miRNA-132 and miRNA-212 protect neurons against H_2_O_2_-mediated cell death, and their loss causes neuronal apoptosis via elevated levels of the cell death-associated proteins PTEN, FOXO3 and P300 that antagonize Akt pro-survival signaling (Wong et al., 2013). Knockdown of miRNA-132 in the hippocampus impairs memory acquisition (Wang et al., 2013), which can be regarded as a marker for cognitive impairment (Xie et al., 2015). The brains of Alzheimer patients demonstrated decreased miR-132/212 level already at stages Braak III and IV of the disease and in a manner related to Tau pathology (Lau et al., 2014). Regarding our data, it may be suggested that neuro-inflammation up-regulates miRNA-132/212 as an anti-inflammatory reaction, which also protects the brain cells from excessive reactive oxygen species toxicity. The α7(1–208)-specific antibodies weaken this reaction but do not decrease miRNA-132/212 below the control level. Obviously, the final pro- or anti-survival antibody effect is an integral interplay of multiple miRNAs and their targets.

The KEGG pathways analysis allowed deeper understanding of miRNA regulating activity in the brain under the effects of LPS, LPS and antibody or nicotine. According to the probability of engagement, the predicted signaling pathways could be classified into three groups (Figure 5A): extremely probable (−log *p* > 20: Axon guidance, MAPK and PI_3_K/Akt pathways); highly probable (20 > −log *p* > 10: TcR, mTOR, Wnt, TGFβ and Neurotrophin pathways) and probable (10 > −log *p* > 2: all other predicted pathways).

The first group includes pathways found under many different receptors; therefore, it is impossible to identify any definite one. However, comparison of −log *p* values found for LPS and LPS + Ab treatments allows suggesting that the antibody contributes additional signaling through these pathways. This is in accord with our experimental data on the ability of α7-specific antibody to stimulate IL-6 production in U373 cells through p38-dependent pathway (Figure 7A). The involvement of both MAPK and PI_3_K/Akt pathways in α7 nAChR signaling is well documented (Parada et al., 2010; Yu et al., 2011; Cucina et al., 2012; Huang et al., 2012; Cui et al., 2013). Previously we reported that signaling of α7 nAChRs expressed in mitochondria can be triggered by α7-specific antibody (Gergalova et al., 2014), therefore, the antibody could engage PI_3_K/Akt pathway in the brain.

The second group contains signaling pathways of TcR, TGFβ, Wnt and Trk (neurotrophin) receptors, as well as mTOR pathway. Again, the antibody contributes to these pathways compared to LPS alone. The maximal increase of −log *p* was observed for TcR (22 vs. 15), mTOR (17 vs. 12) and neurotrophin (10.5 vs. 7.7) signaling pathways. Neurotrophins acting through Trk receptors activate PI_3_K/Akt and MAPK pathways, and the mTOR functions downstream of PI_3_K/Akt signaling pathway in response to cytokines and growth factors (LoPiccolo et al., 2008; Longo and Massa, 2013); therefore, their engagement by the antibody may be explained by the antibody effect found in group 1. Neurotrophin signaling is strongly stimulated by nicotine (14 (Nic) vs. 7.5 (LPS) vs. 10.5 (LPS + Ab)) that also assumes the involvement of nicotinic receptors and, possibly, their cross-talk with Trk receptors. T lymphocytes are normally not found in the brain parenchyma but can penetrate there under neuroinflammation when the blood-brain barrier is disrupted (Engelhardt, 2006). In addition, they could be found in the brain capillaries (since the brains had not been perfused before RNA extraction). The antibodies significantly contributed to this pathway; therefore, they could facilitate T lymphocyte migration to or penetration into the brain and subsequent activation resulting in TcR signaling pathway involvement. The Wnt and TGFβ signaling pathways are engaged by all three treatments; however, there is no significant difference between nicotine, LPS or LPS + Ab-treated mice.

Among other signaling pathways of interest are the Notch and apoptosis signaling, which are found in nicotine- and LPS + Ab- but not LPS-treated mice and, therefore, relate to nicotinic receptors. The Notch signaling pathway components expressed in the brain were shown to be involved in the pathogenesis of AD (Woo et al., 2009) and, therefore, this pathway may contribute to the memory impairment found in α7(1–208)-immunized mice (Lykhmus et al., 2015). The involvement of nAChRs in regulating cell survival and apoptosis has already been discussed. The data of KEGG analysis predict the miRNA-mediated regulation of apoptosis pathway by α7(1–208)-specific antibodies that is in accord with the analysis of molecular targets of miRs 9, 99 and let-7g described above.

LPS notably stimulates pro-inflammatory cytokines production via toll-like receptor type 4 (TLR-4)/CD14 receptor complex, resulting in MAP kinases activation and a nuclear localization of NF-κB (Kawai and Akira, 2010; Nadorp and Soreq, 2015) also engaging TLR-4 to Src activation (Liu et al., 2012). Here, we show that similar enzymes (Src kinases and p38) are involved in the signal transduction pathway from either LPS or α7-specific antibodies, suggesting that the α7 nAChR can directly influence TLR-4 signaling. Together with the parallel TLR9/ACh interaction (Nadorp and Soreq, 2015), these findings explain how ACh may attenuate the LPS-induced pro-inflammatory cytokine production through α7 nAChR. Inversely, α7-specific antibodies stimulate TLR-4 pro-inflammatory signaling, predicting close TLR-4 and α7 nAChR proximity in the plasma membrane. Although LPS failed to prevent binding of the α7-specific antibodies to U373 cells in flow cytometry (data not shown), one might predict an intersection of α7 nAChR signaling with TLR-4 at the level of adaptor proteins or Src-kinases, i.e., at the very upstream, plasma membrane-proximal stage.

**Figure 8:**
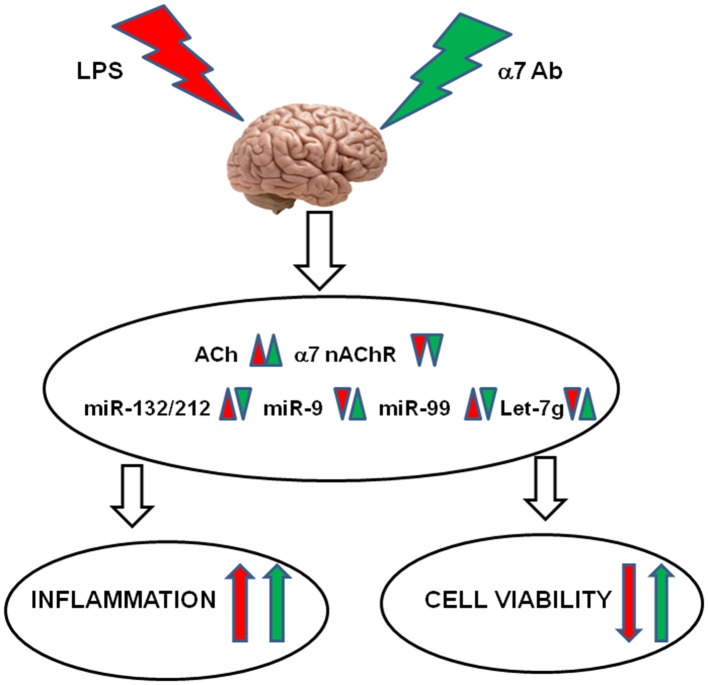
**Schematic diagram demonstrating the effects of LPS and α7(1–208)-specific antibodies (α7Ab) in the brain.** LPS down-regulates AChR expression resulting in acetylcholine increase, down-regulates α7 nAChR expression and changes the levels of miR-132/212, miR-9, miR-99 and let-7g. The α7Ab further decrease AChE and α7 nAChRs but antagonize LPS effects on miRNAs. As a result, the α7Ab aggravates the LPS-induced inflammation but may support the brain cell viability.

Taken together, our current findings, summarized in Figure 8, demonstrate that:

1. LPS-induced inflammation stimulates the ACh-mediated and miRNA-regulated anti-inflammatory pathway in the brain; however, down-regulation of the α7 nAChRs makes this pathway ineffective;
2. Inflammation dampens the mitochondrial cholinergic anti-apoptotic pathway and stimulates miRNAs assumed to decrease the brain cells viability;
3. The α7-specific antibody aggravates LPS-induced inflammation by preventing the expression of anti-inflammatory miR-212 and stimulates the LPS-like signaling by itself;
4. The α7-specific antibody dampens the excessive up-regulation of pro-apoptotic miRNAs upon inflammation and maintains mitochondrial integrity that may support the brain cells viability.

This data support the idea that excessive inflammation is an important pathogenic factor stimulating neurodegenerative processes. In this context, α7 nAChR-specific antibodies may play a dual role: potentiating a certain level of inflammation but preventing its neurodegenerative consequences.

## Author Contributions

MS, SK, and HS: substantial contributions to the conception and design of the work; OL, NM, LK, OK, GG, and KU: the acquisition, analysis, and interpretation of data for the work; MS, NM, and HS: drafting the work; OL, LK, OK, GG, KU, and SK: revising it critically for important intellectual content; OL, NM, LK, OK, GG, KU, SK, HS, and MS: final approval of the version to be published. Agreement to be accountable for all aspects of the work in ensuring that questions related to the accuracy or integrity of any part of the work are appropriately investigated and resolved (OL, NM, LK, OK, GG, KU, SK, HS, and MS).

## Funding

Support of this study by the European Research Council under the European Union’s Seventh Framework Programme (FP7/2007–2013)/ERC Advanced Award 321501 and the Israel Science Foundation grant no.817/13 (to HS) is acknowledged. NM was a recipient of post-doctoral fellowships from the ELSC Brain Center and The Israeli Government.

## Conflict of Interest Statement

The authors declare that the research was conducted in the absence of any commercial or financial relationships that could be construed as a potential conflict of interest.

## Footnotes

^1^http://diana.imis.athena-innovation.gr/DianaTools/index.php?r=mirpath/index
